# Professional identity formation in the transition from medical school to working life: a qualitative study of group-coaching courses for junior doctors

**DOI:** 10.1186/s12909-016-0684-3

**Published:** 2016-06-24

**Authors:** Lydia de Lasson, Eva Just, Nikolaj Stegeager, Bente Malling

**Affiliations:** Department of Anesthesiology and Intensive Care, Aarhus University Hospital, Palle Juul-Jensens Boulevard 100, 8200 Aarhus N, Denmark; Consulting Company Justeva, Aldersrovej 23 D, 8200 Aarhus N, Denmark; Department of Learning and Philosophy, Aalborg University, Kroghstræde 3, 9220 Aalborg Ø, Denmark; Postgraduate Medical Education, Centre for Health Sciences Education, Faculty of Health, Aarhus University, Palle Juul-Jensens Boulevard 82, 8200 Aarhus N, Denmark

## Abstract

**Background:**

The transition from student to medical doctor is challenging and stressful to many junior doctors. To practice with confidence and professionalism the junior doctors have to develop a strong professional identity. Various suggestions on how to facilitate formation of professional identity have been offered including the possible positive effect of group-coaching courses. The purpose of this study was to explore how group-coaching might facilitate professional identity formation among junior doctors in the transition period.

**Methods:**

Group-coaching courses comprising three whole-day sessions and five 2 h sessions during a period of 4 months were offered to junior doctors in the first years after graduation. The purpose was to support the participants’ professional development, ability to relate to patients, relatives and staff and career development. The coaches in this study had a background as health professionals combined with coaching educations. Data was obtained through observations, open-ended questionnaires and interviews. A generic thematic analysis was applied.

**Results:**

Forty-five doctors participated in six coaching groups. The three main themes emerging in the sessions were: Adoption to medical culture, career planning, and work/life-balance. The junior doctors found the coaching intervention highly useful in order to cope with these challenges. Furthermore, the group was a forum where the junior doctors could share thoughts and feelings with colleagues without being afraid that this would endanger their professional career. Many found new ways to respond to everyday challenges mainly through a new awareness of patterns of thinking and feeling.

**Conclusions:**

The participants found that the group-coaching course supported their professional identity formation (thinking, feeling and acting as a doctor), adoption to medical culture, career planning and managing a healthy work/life-balance. Further studies in different contexts are recommended as well as studies using other methods to test the results of this qualitative study.

**Electronic supplementary material:**

The online version of this article (doi:10.1186/s12909-016-0684-3) contains supplementary material, which is available to authorized users.

## Background

A strongly rooted professional identity enables doctors to practice with confidence and professionalism convincing others of their practical abilities [1]. Some researchers have thus suggested that a major focus of medical education should be the development of a professional identity [2–4]. The close relation between professional identity and professional performance demands that newly graduated doctors continuously focus on developing a professional identity while facing the many practical challenges in the transition from medical school to clinical practice. Since the formation of a professional identity requires participation in a professional community [5], medical schools cannot fully prepare students for the life as doctors [6–8]. Thus, reports on how stressful the transition from student to doctor is perceived by junior doctors continue to emerge [9–19].

Previously, research has focused on the importance of creating a supportive and alleviating learning environment [20–24], especially for the newcomers in the profession. The reforms in postgraduate medical education in many countries have improved the learning and teaching methods, and created an increased understanding of the necessity of participation, supervision and feedback to enhance learning [25]. Still, many junior doctors report that the challenges they face exceed their competencies and 25 % have experienced burnout in their first working year [11, 14, 15, 26].

We identified six studies with directions on how to facilitate professional identity formation among junior doctors [2, 4, 14, 17, 19, 27]. A previous study showed that a small group of doctors in the later years of specialist training found new ways of dealing with professional challenges through participation in group-coaching [28]. Group-coaching supported their development of a professional attitude to everyday working challenges and provided a method for them to reflect on and evaluate their own performance [28].

This is well in accordance with the perception of coaching as a mean to unfold a person's potential in order to maximize performance [29]. Many studies have demonstrated the effectiveness of coaching [30–32]. Within the medical field, coaching has contributed to increased wellbeing and reduction of stress and burnout [33–36]; moreover, it has supported competence building [37–39]. However, there are no reports on the use of group-coaching to facilitate the transition period for junior doctors. The purpose of this study was to explore the use of group-coaching in the first working years to support development of professional identity in the transition from medical school to working life as a doctor.

The research question was: How do junior doctors attending group-coaching describe the challenges they face regarding professional identity formation in the transition from student to doctor and do they consider coaching a helpful tool to develop a professional identity as a doctor?

## Methods

### Participants

Junior doctors in their first working years were invited by mail to participate in a group-coaching course. Participants were included on a first come first served basis. To promote a culture characterised by honesty and freedom of speech, the participants in a group were not employed in the same department. The participants gave informed consent and anonymity was guaranteed. Ethical approval was not required for this type of study in our jurisdiction.

### Intervention

All participants attended a full coaching course of three whole-day sessions and five 2 h sessions during a period of 4 months. Coaching was based on a systemic perspective [40]. The purpose of the coaching was to support the participants’ professional development, their ability to relate to patients, relatives and staff and their career development. The coaches were one medical consultant and two former nurses. Each course was run by the doctor and a nurse. The medical consultant had a Masters’ degree in Organizational Coaching. The nurses had academic degrees. One held a PhD in learning processes; the other a diploma in coaching from Cambridge University.

Prior to the course, the participants were asked to define individually which professional challenges they intended to work with in the course. Every session included a theoretical theme (e.g., conflict management, systemic coaching theory etc.), and a group-coaching session. Participants were encouraged to read selected material in relation to these theoretical themes. A typical group-coaching session rested upon a dialogue between the coach and the person being coached (the coachee) interrupted by requested input from the group. The group gave support to the coachee by reflecting his/her words or recognizing the efforts of the coachee. Each participant took the position of a coachee at least once during the course.

### Data collection

#### Evaluation of the coaching course

The research group developed the evaluation forms. Comments from the first group on the evaluation forms were incorporated in the final version consisting of five and ten open questions, respectively. The forms are presented in Appendices 1 and 2 [see Additional file 1]. Halfway through the course and immediately after the last session participants filled in the evaluation forms. The former of these evaluations was supplemented by a semi-structured group interview. The interviews were tape-recorded and transcribed verbatim.

#### Observations

In each session one of the two coaches made observations and recorded conversational themes, important comments, interventions and own reflections.

#### Data analysis

A generic thematic analysis was applied [41]. The researchers independently went through data from the first group to identify common themes. Upon the initial and separate categorisation the researchers through iterative discussions arrived at consensus concerning common themes. All four researchers scored all material independently. Themes and categories relevant for this study are shown in Table 1. The quotes presented in the results’ section are carefully selected to represent general themes. A pseudonym is applied following each quote.

**Table 1:** Analysis – themes, categories and examples

Themes	Categories	Examples
Professional identity formation	Take ”on” and “off” the white coat	How to manage the huge responsibility – and how to leave the problems when leaving work
	Stress and recovery	How to manage the many stressful incidents (death, sick children, many rotations, night shifts)
	Empowerment	How to be able to react and act in a more proper (constructive) way
	Reflections on relations (new or altered perspectives)	Learn to acknowledge that other people have other perspectives
	Self-confidence	Feeling incompetent
	Self-efficacy	Afraid of making mistakes or wrong decisions
Career planning	Career choice and planning	How do I plan to reach my wish regarding specialty? Do I dare to go for this specialty?
Work/life balance	Work/life balance	How to deal with my own ambitions of being perfect both at work and at home

Table 1 shows the three main themes young doctors presented in a group-coaching course. Themes are categorized and illustrated by anonymized examples

## Results

A total of 45 doctors participated in the six coaching groups (38 female, 7 men).

Below the results are presented with two main headings: “Professional identity formation” and “Evaluation of the coaching intervention”. The three major themes emerging from the coaching sessions regarding professional identity formation were: 1) “Adoption to medical culture”; 2) “Career planning” and 3) “Work/life-balance”.

### Professional identity formation

#### Adoption to medical culture

*“It is difficult to have a smooth transition from medical school to the job as a doctor. There are so many elements in the transition, which you cannot learn in medical school. As a doctor you suddenly carry an immense responsibility and you are under severe pressure physically as well as psychologically”*

(Cathy, 1^st^ year in Hospital Setting)

Many of the junior doctors attending group-coaching came to share their early work experiences, and their concerns about what kind of doctor they were about to become. Many participants experienced stress-related symptoms due to the unpredictable nature of their work, the heavy workload and the constantly changing demands. A general theme that often came up during the sessions was the junior doctors’ reluctance to embrace the responsibilities of being a doctor and thus running the risk of making mistakes. Decision-making was experienced as a heavy burden due to their lack of experience and varying support from senior doctors. Many considered patients better served by a specialist doctor and felt inferior in comparison to other doctors. The pattern of hard self-criticism and lack of self-confidence was a topic in all groups. The problems often arose from a belief that, as educated doctors, they should be able to handle the complicated situations at work. Thus, their feelings of insecurity and self-doubt were regarded as a lack of professional competence.

Their feelings were aggravated by not having anybody to share these thoughts with at work and many experienced loneliness. In some cases the overwhelming feeling of responsibility and the lack of support caused the junior doctors to consider leaving the profession.

*“You work so much alone, which makes you think that you are the only one who thinks that it is tough”.*

(Kamilla, 2^nd^ year in Hospital Setting)

#### Career planning

Most participants were concerned about choice of medical specialty. Many thought that they were expected to be able to identify the “only” specialty right for them. When struggling to identify a “perfect career path” many tended to interpret this as another proof of their professional incompetence. Other participants were to a higher degree certain about their professional aspirations, but lacked the courage to pursue these ambitions – often due to fear of hard competition and low self-confidence.

Considerations on career choices usually included their family situation. Spouses were often busy and ambitious too, and most of them not able to move to other parts of the country, which for some participants was necessary to pursue their wish for a certain medical specialty. The women were devoted to motherhood as well and often stated that their family had first priority, realising that this would impact on their career options and choices.

*“The good life is family life – time – preferably four children, but also work and be a good doctor – is that possible?”*

(Karin, 1^st^ year in Hospital Setting)

#### Work/life-balance

*“I was beginning to feel that my professional career was taking control over my entire life. I realized that I signed up for this course hoping that this would be my rescue”*

(Jennifer, 2^nd^ year in Hospital Setting)

Quite often, the participants had problems leaving stressful and anxiety-provoking events behind when off work. Typically, these problems referred to the burden of responsibility felt by the doctors when treating acutely or severely ill patients. Other events were distressing communication with colleagues, other staff, patients or relatives. The junior doctors stated that even though the matter had been dealt with in a satisfactory manner, the matter often stayed on their mind after work leaving them with impaired recreation or sleep disturbance.

Many of the junior doctors were very ambitious and many felt that their families too had high expectations to them. They were among the top students and were used to success in academia. They dreamt of applying for a prestigious post while at the same time striving for perfection in their family life. A common theme in the coaching sessions was that many felt they had to either lower their career ambitions or their ambitions about the “good” family life.

*One participant reported her daily life schedule this way: 50 % of her time at work, 25 % was reserved for sleep, 15 % on recreational activities and friends and the last 10 % on activities to improve her qualifications. She found that this distribution served her well at the time being but worried what to do if she was to have children. She wondered if she could reduce sleeping time to create more time for family activities.*

(Mary, 2^nd^ year Hospital Setting, Coaches’ note from a coaching session)

### Evaluation of the coaching intervention

Coaching was seen as a major contributor to the development of the junior doctors’ professional identity. In their final evaluation the participants typically stated that they had gained a new awareness of their patterns of thinking, feelings and reactions and found new ways of taking control of their professional lives.

The participants felt more at ease with themselves, making the stressful transition period endurable. Many felt that this was actually the most important outcome of the intervention.

*“Through coaching I have become more aware of myself, my behaviour, how to handle a situation and alternative ways of thinking and acting. This has helped me to become more balanced in different situations and in my professional role. I came for good advice. Gradually I understood that it is my own responsibility and regained the control over my life”.*

(Gretha, 1^st^ year in Hospital Setting)

The participants found the group setting highly supportive. The meeting with peers served as an invaluable experience. Listening to the stories of others made everybody recognize that insecurity and fear of making mistakes is a part of every junior doctor’s transition from student to doctor. This was a relief to many, who had considered themselves weak.

*“It is a relief to find out that others feel the same way as I do. I now feel assured that although I have difficulties carrying the professional responsibility, I am not a bad doctor”.*

(Frederikke, 2^nd^ year in Hospital Setting)

It was essential for the participants that none of them worked in the same department at the time of the coaching intervention. Participants allowed themselves to reflect in ways they felt might have endangered their career had there been participants from their own department, and they enjoyed to include more private matters in the conversations.

*“There is a lot of competition in my preferred specialty and I would not feel so comfortable if my colleagues (from the job) were in this group. In that case I would not have let down my guards the way I have”.*

(Carl, 2^nd^ year in Hospital Setting)

The coaches being health professionals meant that the participants did not have to explain critical clinical settings or outline cultural issues in the health system. They could concentrate on thoughts, feelings and reactions in the coaching sessions. Furthermore, many of the participants indicated that one of the reasons why they were allowed to join the coaching course as a part of their job could probably be assigned to the coaching being conducted in a health-professional setting.

## Discussion

Our study raises several questions regarding junior doctors’ professional identity formation during the transitional period and group-coaching as a supportive method in this respect.

### Professional identity formation

Overall, the participants found that the group-coaching course supported their professional identity formation (thinking, feeling and acting as a doctor), adoption to the medical culture, career planning and managing a healthy work/life-balance. The participants faced exactly the same challenges, previously described in the literature [42–45]. The coaching intervention helped participants reflect on “who they were, who they were becoming and who they wished to become” [2]. Cruess & Cruess [2] argue that students should be supported in this process and that medical schools ought to devote more attention to the process of professional identity formation. In accordance with previous research [45], our research suggests that junior doctors in the process of moving from legitimate peripheral participation to full participation as medical practitioners [46] also profit from a supportive environment concerning professional identity formation. In this respect, we would like to expand on this view of Cruess & Cruess. Even though training in identity formation at medical school most certainly will help students’ adjust in their medical career, we believe that the focus on identity formation needs to be extended into the transition period – and probably further. You can teach students about the challenges ahead of them going from students to professional doctors and the techniques to apply when dealing with these challenges, but students need to experience how they will respond to these challenges or what it will mean to them when they become doctors. Therefore, we believe the medical profession may profit from an increased focus on identity formation in the transition period. A limited initiative (as the coaching course in this study) can be of quite substantial importance for junior doctors during the stressful period of transition.

### The coaching intervention

Traditionally, coaching within the medical field has been carried out as individual sessions [34, 47, 48]. This might be a preferable strategy for more experienced doctors. However, our research suggests that group-coaching is a very effective method of supporting junior doctors’ professional identity formation during the transition period due to the sharing of common experiences. In our study most participants preferred group-coaching compared to individual courses, probably due to the comforting realization of not being the only one struggling. This is in accordance with Stelter et al. [49], who found that group-coaching interventions had a significant effect on the scores for social recovery (a return to effective social functioning following a stressful or traumatic event) and general wellbeing among young sports talents who wished to integrate their sports careers, educational demands and private lives. Furthermore, group-coaching supports development of durable social networks and it increases social capital [50]. However, these positive outcomes are by no means guaranteed. Thus, in this particular study special consideration was taken to form stable and prosperous groups with representatives from different departments. There was no turnover in the groups, and attendees were urged to show up at all sessions. Finally, everybody agreed on confidentiality to ensure a comfortable and confidential atmosphere between attendees – an experience previously unknown to most attendees in collegial settings.

A common debate in coaching research is whether the coach should be an expert on the specific topic or just an expert on coaching [51, 52]. However, in this concrete case, almost all participants stated that it was of utmost importance to the success of the course that the coaches were experienced health professionals. The professional background of the coaches entailed a thorough understanding of the challenges faced by a junior doctor. This prompted a free and active conversation but also allowed the coaches to break out of the traditional coaching position in order to share experiences from their own health professional careers. The professional background of the coaches allowed them to acknowledge the insecurity that many of the participants felt regarding patient treatment – but also regarding career choice. In this light we recommend that coaches working with junior doctors during the transitional period have a deep knowledge and understanding of the healthcare system, and preferably be health professionals themselves.

### Limitations of the study and further research

Since the coaches administered the questionnaires and conducted the interviews, bias might have been introduced due to the bond between coaches and participants. Participants with a wish to specialize in anesthesia might be uncomfortable voicing any criticism at the prospect of possibly working in the same department as one of the coaches. Furthermore, the qualitative approach primarily informs us of the experiences of the participants, but it does not allow for generalization or conclusions about the sustainability of the effects reported by the participants. In addition, it is unknown whether the participants in the coaching course were representative for junior doctors in general regarding presentation of their professional and personal problems. In an attempt to address some of these methodological problems we have initiated a longitudinal study following two groups of newly graduated doctors (*n* = 400) for 18 months (about 5 % participated in the coaching course). We hope this study will confirm the results in this article, but we also urge other researchers to repeat the study in other contexts.

## Conclusions

Junior doctors found adoption to medical culture, career choice and work/life balance challenging. Through group-coaching participants found new ways of dealing with everyday challenges and learned to use peer discussions to disclose uncertainty and doubt without the fear of being regarded as less competent. In this respect, group-coaching might help to facilitate the transition from medical student to doctor. A prerequisite for the outcome of the study was the lack of professional competition between group members and a stable and comforting environment within the group. The legitimacy of participation was promoted by the coaches being experienced health professionals and trained coaches.

## Additional file

Additional file 1: Evaluation forms. Description: Midway and end of course evaluation forms. (DOCX 21 kb)
