# Supplementary material for: Professional identity formation in the transition from medical school to working life: a qualitative study of group-coaching courses for junior doctors
Source: BMC Med Educ. 2016 Jun 24;16:165. doi: 10.1186/s12909-016-0684-3 (PMC4919855; doi:10.1186/s12909-016-0684-3)
Supplement: Additional file 1: — Evaluation forms. Description: Midway and end of course evaluation forms. (DOCX 21 kb) [file 12909_2016_684_MOESM1_ESM.docx]

# Evaluation forms

| **Appendix 1**  **Midway evaluation form** |  |
| --- | --- |
| What does it mean to you to participate in this group-coaching course? |  |
| What does the peer community and the experiences reported by the other participants mean to you? |  |
| How has participation in this group-coaching course influenced your daily working life (please give specific examples)? |  |
| How do you think that other people (superiors, peers, patients, your relatives) have noticed that you are participating in this course? |  |
| Have you received feedback? |  |

| **Appendix 2**  **End of course evaluation form** |  |
| --- | --- |
| What was your primary focus, and how will you describe your expectations to the group-coaching course in relation to this focus? |  |
| To which extent were your expectations fulfilled? (0 = not at all; 10 = totally fulfilled) |  |
| How has group-coaching affected your ability to act in relation to your primary focus? |  |
| How has participation in the group-coaching course influenced your reflection and interaction with patients and relatives? |  |
| How has participation in the group-coaching course influenced your reflection and interaction with colleagues in the department? |  |
| How has participation in the group-coaching course influenced your reflection and interaction with other collaborative partners? |  |
| How has participation in the group-coaching course influenced your career planning? |  |
| What were the implications of participants coming from different departments? |  |
| How will you describe the importance of being in a group regarding your benefit from participation in the course? |  |
| Please rate the importance of the different elements of the course (0 = no importance; 10 = very important):  Group-coaching  Theoretical presentations  Reading theoretical articles  Informal networking |  |
